# Development of an Embedded Myokinetic Prosthetic Hand Controller

**DOI:** 10.3390/s19143137

**Published:** 2019-07-17

**Authors:** Francesco Clemente, Valerio Ianniciello, Marta Gherardini, Christian Cipriani

**Affiliations:** 1 The Biorobotics Institute, Scuola Superiore Sant’Anna, 56127 Pisa, Italy; 2 Department of Excellence in Robotics & AI, Scuola Superiore Sant’Anna, 56127 Pisa, Italy

**Keywords:** hand prosthesis, embedded control system, magnetic sensors, passive magnetic markers, myokinetic controller

## Abstract

The quest for an intuitive and physiologically appropriate human machine interface for the control of dexterous prostheses is far from being completed. In the last decade, much effort has been dedicated to explore innovative control strategies based on the electrical signals generated by the muscles during contraction. In contrast, a novel approach, dubbed myokinetic interface, derives the control signals from the localization of multiple magnetic markers (MMs) directly implanted into the residual muscles of the amputee. Building on this idea, here we present an embedded system based on 32 magnetic field sensors and a real time computation platform. We demonstrate that the platform can simultaneously localize in real-time up to five MMs in an anatomically relevant workspace. The system proved highly linear (*R*^2^ = 0.99) and precise (1% repeatability), yet exhibiting short computation times (4 ms) and limited cross talk errors (10% the mean stroke of the magnets). Compared to a previous PC implementation, the system exhibited similar precision and accuracy, while being ~75% faster. These results proved for the first time the viability of using an embedded system for magnet localization. They also suggest that, by using an adequate number of sensors, it is possible to increase the number of simultaneously tracked MMs while introducing delays that are not perceivable by the human operator. This could allow to control more degrees of freedom than those controllable with current technologies.

## 1. Introduction

The loss of a limb, e.g., caused by vascular/infectious diseases or trauma, is an event that strongly affects the quality of life under several aspects. Indeed, this event usually limits the person in performing working and daily living activities, thus having a strong impact on the social life. To date, an upper limb prosthesis able to achieve the dexterity (22 degrees of freedom–DoFs) and the grasp force (more than 500 N) of the human hand with anatomical size and weight (about 400 g) is far from being realized.

Focusing on the control strategy of such devices, commercially available artificial hands and arms exploit surface electromyographic (EMG) electrodes that record the electrical activity generated by the residual muscles during contraction. When independent agonist/antagonist muscle pairs are available each of these can be mapped to a unique function in the prosthesis, thus implementing the so called direct control [1]. The latter is the most intuitive and robust approach [2], albeit either not possible, due to the lack of accessible independent control sources with surface electrodes or inefficient [3] in the case of multiple functions/DoFs.

To overcome this limit and enhance the number of naturally controllable DoFs, several alternatives driven by technological innovations and surgical procedures were proposed. Novel technologies allowed direct interfacing with the physiological structures involved in motor control, e.g., wireless implantable myoelectric sensors (IMES) [4,5] or epymisial electrodes wired through osseointegrated implants [6,7,8]. In a complimentary fashion, new surgical procedures, like the targeted muscle reinnervation [9] or the regenerative peripheral nerve interfaces [10] made possible the creation of novel control sites.

In this work, we focus on an alternative solution for the control of dexterous prostheses first introduced by our group, and dubbed the myokinetic approach [11]. The idea behind this method is to implant permanent magnets (magnetic markers—MM) into the residual muscles, track their movements using magnetic sensors, and use these signals as control inputs in a prosthesis. As an example for transradial amputations, MMs would be implanted in the residual forearm muscles, i.e., the extrinsic muscles of the hand. Knowing the position of each marker implanted in a specific muscle would then allow to control the DoF of the prosthetic hand originally controlled by that muscle. Indeed, localizing the position of the magnet is equivalent to measuring the contraction/elongation of the muscle it is implanted in, as the magnet moves with it. If successful, this approach could allow to physiologically (i.e., simultaneously and proportionally) control multiple, independent DoFs of the prosthesis by exploiting simple passive implants.

In our previous work, we presented a system able to localize online up to four MMs using six magnetic sensors and a desktop PC [11]. Building on this, we developed and validated an embedded version able to retrieve online the pose (i.e., the position and orientation) of up to five MMs using 32 sensors. We assessed its performance using a 3D positioning platform and an anatomical mockup of the human forearm with simulated muscles. In particular, the 3D positioning platform was used to evaluate the accuracy and precision of the localization with respect to the distance between the sensors and one single magnet. The mockup was instead used to simulate muscle movements along anatomical trajectories in order to compare the performance (accuracy, precision and computation time) between the embedded solution and the PC version.

The results with the 3D platform provided insights on the maximum distance between sensors and the implanted magnet. The tests with the forearm mockup demonstrated a localization error in the range of ~10% the entire trajectories, remarkable precision (1% repeatability) and high linearity (*R*^2^ = 0.99), for both setups. The embedded system, however, proved four times faster than the PC implementation, exhibiting a computation time below 4 ms for localizing up to five MMs. These outcomes demonstrate the feasibility of implementing simultaneous, independent and continuous control of individual digits on a hand prosthesis using the myokinetic approach on a portable device.

## 2. Materials and Methods

### 2.1. Architecture

**Figure 1:**
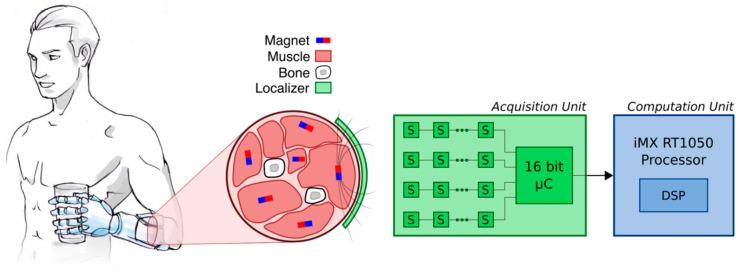
Embedded system architecture. Overview of the embedded myokinetic control interface for a prosthetic hand. Permanent magnets are implanted in relevant residual muscles and their magnetic field is acquired by a matrix of sensors (“S” on the acquisition unit) in order to compute (computation unit) their position. In the target application—in a prosthetic hand—this information could be used to control its movements in physiologically appropriate manner.

Generally speaking, a myokinetic control interface comprises multiple MMs, each one implanted in one target muscle, and a localizer hosted in the prosthetic socket ideally constrained to the skin (Figure 1). The localizer is responsible for continuously estimating the pose of the implanted MMs, using three-axis magnetic field sensors and solving the so-called inverse problem of magnetostatics. The computed pose vector is then used as a control input for the prosthesis [11]. As the inverse magnetostatics problem does not have a closed-form solution, a numerical approximation is required. Hence, in a representative implementation, the localizer numerically derives the poses of N MMs by modelling the three-axis field at the *i*th sensor, *B_i_*, as a linear superposition of that produced by N magnetic dipoles [11]:

(1) $$B_{i}=\overset{N}{\underset{j=1}{\sum }}\frac{M_{j}\mu_{r}\mu_{0}}{4\pi }\left(\frac{3\left(\hat{m_{J}}\cdot x_{ij}\right)x_{ij}}{{\left|x_{ij}\right|}^{5}}-\frac{\hat{m_{J}}}{{\left|x_{ij}\right|}^{3}}\right)$$

where *M_j_* and *m_j_* are the magnitude and the direction of the magnetic moment of the *j*th MM, respectively, and *x_ij_* is the vector distance between the *i*th sensor and the *j*th MM. A number of equations equal or higher than 6 N (viz. a number of three-axis sensors ≥ 2 N) is needed to retrieve the unknown poses (*x*, *m*).

In this work we present an embedded version of the localizer, which is composed of two main parts: (i) An acquisition unit (AU) containing a grid of 32 sensors that samples the field generated by (up to) five MMs; and (ii) a microprocessor-based computation unit (CU) that processes the signals and continuously retrieves the poses of the MMs (Figure 1).

**Figure 2:**
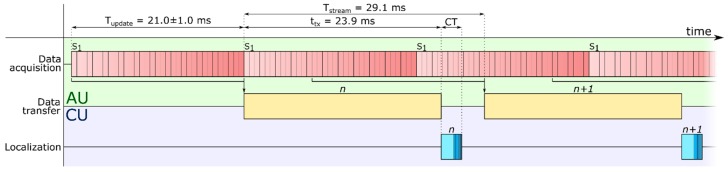
Temporal diagram of the tasks involved in the embedded localizer. The acquisition unit (AU) continuously samples the data from the sensor matrix (21.0 ± 1.0 ms update period) and transfers (taking 23.9 ms) the latest acquired data package to the computation unit (CU) every 29.1 ms, and subsequently starts acquiring the *n*th + 1 package. The CU localizes each data package in CT (computation time) seconds.

The AU consists of a custom board containing 32 three-axis magnetic field sensors (MAG3110, NXP Semiconductors NV, Eindhoven, Netherlands; full-scale output of ±10 G and sensitivity of 1 mG), placed in a 4 × 8 matrix (9 mm inter-sensor distance). All the sensors are connected to a 16-bit architecture microcontroller (dsPIC33EP512MU810-I/PT, Microchip Technology Inc., Chandler, AZ, USA). The latter sequentially samples the sensor readings (with a variable lag across samples of 21.0 ± 13.5 ms) and transmits them to the CU at a fixed period (29.1 ms), over an UART RS-485 communication bus (Figure 2). The transfer time for the whole data package containing the 96 magnetic field data (3 axes × 32 sensors) is 23.9 ms. The power consumption of the AU is 550 mW.

The CU consists of a commercial evaluation kit (MIMXRT1050–EVKB from NXP Semiconductors NV, Eindhoven, Netherlands [12]), which is based on the i.MX RT1050 Real Time Processor that runs on an Arm Cortex-M7 core at 600 MHz (430 mW power consumption). For each new data package, the CU implements the Levenberg–Marquardt algorithm (LMA) to solve the system of 96 Equations (1), taking a certain computation time (CT). The LMA is a widely-used iterative optimization procedure [13,14] that in the present implementation, searches for the solutions that minimize the squared difference between the magnetic field measured by the sensors and that analytically modelled by Equation (1). The LMA was chosen as it was found to outperform other optimization algorithms in terms of localization performance and computation time [13]. Specifically, the LMA implementation from the freely available CMinpack library was used [15]; this is a light version of LMA, not requiring for an operating system and thus suitable for an embedded solution. The computation time of the LMA cannot be predicted a priori since the number of iterations needed to converge to a solution depends on the unknown configuration of the magnets in space. However, special attention was taken to speed up such a computation time. In particular, at each iteration, the Jacobian matrix was analytically computed, rather than being numerically approximated, in order to maximize the efficiency of the algorithm [16]. In addition, most of the code was parallelized by exploiting the DSP (digital signal processing) instructions for SIMD (single instruction multiple data) operations supported by the processor [17]. Finally, the result of the LMA for the *n*th data package was used as the initial condition for the *n*th + 1 iterative procedure, after being checked for consistency (i.e., magnets belonging to the workspace and non-overlapped).

### 2.2. Experimental Procedures

The embedded localizer was assessed in terms of its accuracy and precision, using a 3D positioning platform and an anatomical mockup of the muscles in the human forearm. Neodymium cylindrical magnets (*d* = 4 mm; *h* = 2 mm) with axial magnetization (*M* = 0.0254 A·m^2^ and *B_r_* = 1.27 T) were used, as in [11].

The accuracy and precision are known to be affected by approximations in the dipole model, by those introduced when solving the inverse magnetostatics problem numerically, and by the reliability of the sensing apparatus [18]. These may cause errors in estimating the displacement of single MMs (*e_m_*) and can yield to false predictions of simultaneous displacements (i.e., cross-talk—*e_ct_*) in the case of multiple MMs. Thus, if environmental factors are neglected [11,19], position errors (*E_d_*) related to a certain pose *p_x_* (and similarly for orientation errors, *E_o_*), are given by:

(2) $$E_{d}=D_{est}-D_{act}\approx \bar{e_{m}\left(p_{x}\right)}\pm S_{m}\left(p_{x}\right)+\overset{n-1}{\underset{j=1}{\sum }}\left(\bar{e_{\mathrm{ct}\;J}\left(p_{x}\right)}\pm S_{ct}\left(p_{x}\right)\right)$$

where *D_est_* is the estimated position displacement, and *D_act_* is the actual value; *S* describes the variability of the measurements, which affect the estimation of *e_m_* and *e_ct_*. The four contributions in Equation (2) quantify the accuracy (i.e., $\bar{e_{m}\left(p_{x}\right)}$ and $\bar{e_{\mathrm{ct}\;J}\left(p_{x}\right)}$) and the precision ($S_{m}\left(p_{x}\right)$ and $S_{ct}\left(p_{x}\right)$) of the myokinetic localizer. A detailed description of Equation (2) can be found in our previous study [18].

**Figure 3:**
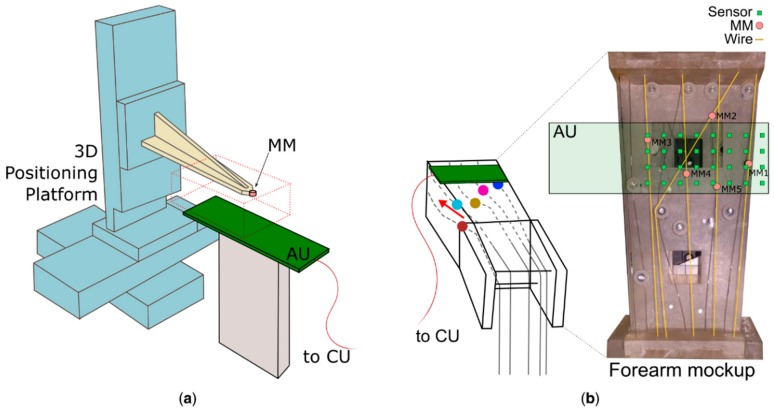
The experimental setup. (**a**) The acquisition unit (AU) samples the magnetic field produced by a single magnet (MM) mounted on a 3D positioning platform, across a ~85,000 mm^3^ workspace. (**b**) The AU samples the field produced by five magnets anchored on the simulated muscles on the forearm mockup (Table 1). The computation unit (CU) is not shown.

A 3D positioning platform (VT-80, PI miCos, positioning precision of 10 µm, Figure 3a) was used to assess the dependency of the accuracy and precision on the distance between the sensors and one MM. The platform was used to move a single MM across a 3D matrix of 11 × 20 × 8 equidistant points (4 mm step). These points were located in a volume centered on the main axes of the sensor grid and extended from 12 mm up to 40 mm above the AU (Figure 3a).

The orientation of the MM was kept fixed, with the magnetization vector oriented perpendicularly with respect to the plane of the sensors. For each point, the magnet was localized 120 times and the accuracy ($\bar{e_{m}}$) was computed for both position and orientation as the mean distance between the estimated pose and its true value. The precision ($S_{m}$) was assessed as the standard deviation of the estimated positions and orientations across the 120 measurements.

An anatomically relevant forearm mockup, equipped with the AU, was used to assess the capability of the system in localizing multiple MMs, akin to our previous work [11] (Figure 3b). The mockup consists of 17 independently controlled wires that run within a parallelepiped workspace through idle pulleys. The wires represent the extrinsic hand muscles as they are routed and can translate in a way to mimic the anatomical position, orientation and physical contraction of the forearm muscles. The embedded localizer was tested in five experimental conditions (viz. configurations), each characterized by a different number of MMs (from one to five) “implanted” into the muscles (i.e., anchored to the wires) (Table 1). In all configurations, one magnet was moved at a time along a 12 mm trajectory defined by the wire in steps of 1.2 mm (11 check-points in total), while the others were kept fixed in their position. The length of the trajectory was derived from anatomical measurements [20]. 100 samples were acquired by the AU for each static spatial configuration of the MMs. These values were fed to both the CU and a PC (Intel i7-6700 CPU running at 3.4 GHz, 32 GB of RAM, Windows 7) in order to compare the performance of the embedded vs. the desktop solution. Specifically, the sampled data were processed on-line by the CU, and transferred to the PC for off-line processing using an implementation of the LMA running on MATLAB R2018b (MathWorks, Natick, MA, USA).

For both the CU and the PC, the $\bar{e_{m}}$ was computed as the average difference between the actual and the estimated displacement of the moving magnet at each step along the entire trajectory. $\bar{e_{ct}}$ was derived for each of the non-moving MMs with the same procedure, by considering a null actual displacement. Finally, the precision components, $S_{m}$ and $S_{ct}$, were derived as the standard deviations of $e_{m}$ and $e_{ct}$ averaged across the 11 trajectory check-points. Although the orientations of the MMs were computed, the orientation errors were not since the actual rotation of the MMs could not be properly controlled and measured along the trajectory.

Finally, besides accuracy and precision, the CT was also assessed and compared between the embedded and desktop localizers. A dedicated timer was used by the CU to measure the CT in the embedded platform (10 μs resolution); stopwatch functions were used in the MATLAB implementation.

## 3. Results

**Figure 4:**
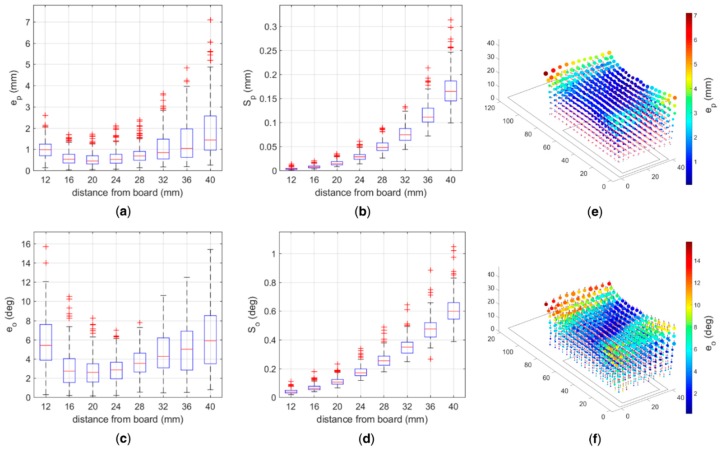
Dependency of the pose accuracy and precision with the distance between the grid of magnetic sensors (3D positioning platform) position accuracy (**a**), position precision (**b**), orientation accuracy (**c**) and orientation precision (**d**) displayed w.r.t. the board distance, in boxplots. The 3D scatterplots show combined (yet qualitative) views about the position accuracy and precision (**e**) and of the orientation accuracy and precision (**f**) in the volume above the board.

The tests with a single magnet using the 3D platform showed that the accuracy of the pose estimation has a non-monotonic trend with respect to the distance between the magnet and the sensors plane (Figure 4a). In particular, the position error ($\bar{e_{m_{p}}})$ ranged between ~0.5 mm at a distance of 20 mm and ~1.5 mm at 40 mm, in its median value; the precision ($S_{m_{p}}$) proved very good and ranged between 14 μm and 165 μm (Figure 4b). Similarly, the mean orientation errors ($\bar{e_{m_{o}}})$ ranged between ~3° at 20 mm and ~6° at 40 mm (Figure 4c), with a precision ($S_{m_{o}}$) spreading between 0.04° and ~0.6° (Figure 4d).

**Figure 5:**
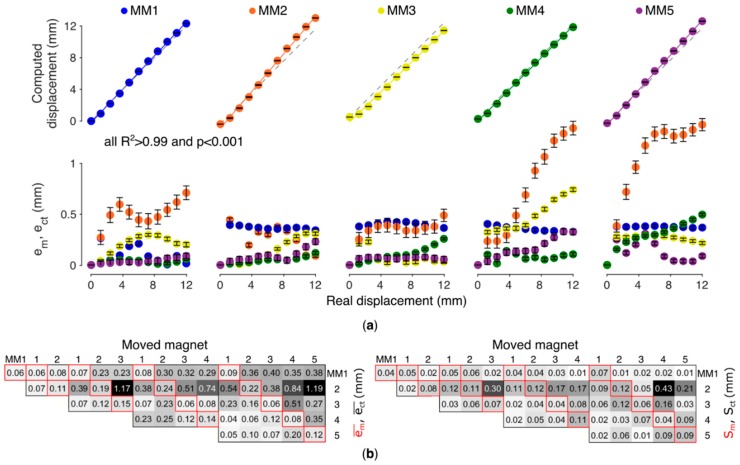
Performance of the localizer under anatomical conditions (forearm mockup). (**a**) Accuracy ($\bar{e_{m}}$ and $\bar{e_{ct}}$) in retrieving the (position) displacement of the magnets for a representative configuration (five MMs, 32 sensors). Each MM was moved along a trajectory of 12 mm, while the remaining MMs were kept at their initial/rest position. Error bars indicate *S_m_* or *S_ct_*. (**b**) Metrics ($\bar{e_{m}}$, $\bar{e_{ct}}$, *S_m_* and *S_ct_*) averaged across the trajectory check-points for each tested configuration of magnets (from one to five). The figures in the diagonals (in red) represent the single magnet accuracy ($\bar{e_{m}}$—left panel) and precision (*S_m_*—right panel); those in non-diagonal positions represent the cross-talk contributions ($\bar{e_{ct}}$—left; *S_ct_*—right).

The embedded implementation of the localizer proved functional also when using the forearm mockup, exhibiting accuracies and precisions comparable to those of the desktop implementation of the localizer (absolute Euclidean difference between the localized poses below 10 µm, for each datum). Hence, only the results from the embedded platform are shown (Figure 5).

The localized displacements and the metrics with the five-magnet configuration are first presented as a representative outcome (Figure 5a). The $\bar{e_{m}}$ ranged between 10 and 380 µm across magnets (Figure 5a). The relationship between the actual and the computed displacement proved highly linear for all MMs (*R*^2^ > 0.99, *p* < 0.001, Figure 5a). The movement of one MM affected the position estimate of the other MMs (due to cross-talk); the importance of this effect varied according to which MM was moved. The maximum $\bar{e_{ct}}$ (1.38 mm, corresponding to ~11% the length of the trajectory) was measured on the position estimate of MM2 while moving MM5.

The mean $\bar{e_{m}}$ (averaged across the trajectory) proved always lower than 220 µm. This corresponds to less than 5% the entire trajectory of each magnet. The mean $\bar{e_{ct}}$ proved lower than 1.19 mm, i.e., less than 10% the entire stroke. The mean $S_{m}$ ranged between 40 µm (MM4) and 120 µm (MM2). The mean $S_{ct}$ was maximum for MM2 (and equal to 430 µm), caused by the movement of MM4. In fact, MM2 proved the magnet with higher sensibility to the movements of the other magnets (i.e., higher cross-talk) and to the variability associated to repeated measures. Conversely MM5 proved the most immune to such effects. Accuracy and precision did not substantially change with a smaller number of magnets with MM2 always being the most sensible (Figure 5b).

**Figure 6:**
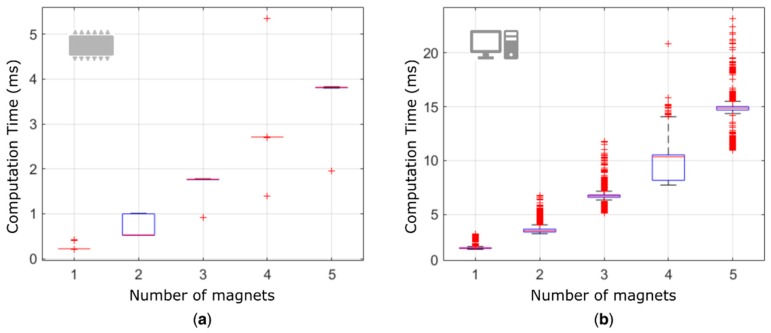
Computation times. CT in retrieving five magnets using the forearm mockup using the embedded (**a**) and desktop (**b**) implementations.

Given the linear superposition of the effects in Equation (1), the computation time linearly increased (*R*^2^ ~ 95%) with the number of magnets, for both the embedded and the desktop implementation, as expected (Figure 6). The embedded platform was able to localize 5 MMs (positions and orientations) in less than 4 ms, compared to 15 ms required by the desktop implementation. On average, a ~75% decrease in computation time was observed with the embedded platform. Notably, the real-time engine of the CU allowed the embedded platform to be much more repeatable (lower variance in the CT).

## 4. Discussion

In this work, a myokinetic localizer for the eventual control of a hand prosthesis was implemented on an embedded real time system and experimentally assessed using a 3D platform and a forearm mockup. The performance, in terms of accuracy and precision with up to five magnets, were comparable to those achieved on a desktop PC implementation, while the computation time proved largely reduced.

The results obtained with the 3D platform confirm that the errors increase with distance [18] and suggest to place the magnets within 36 mm from the plane of the sensors. Indeed within such height the median position error ($\bar{e_{m_{p}}})$ is lower than 1.1 mm (Figure 4), i.e., ~10% the range of motion of extrinsic hand muscles [20], which can be considered as the upper bound of the localization error. In addition, the median precision ($S_{m_{p}}$), which is the most relevant metric to measure the performance of the myokinetic localizer [11], is below 120 μm. In fact, the functionality of the system is not associated with the ability to retrieve the absolute displacement from the rest position, but on the repeatability of the measurement.

The non-monotonic trend of the position and orientation accuracies with distance is in contrast with the results from similar studies [18]. Perhaps this may be caused by un-modelled ferromagnetic components on the AU board or inside the sensors that may either deflect the magnetic field (making the assumptions behind Equation (1) even more approximated) or produce a remnant magnetization that yields to an offset in the readouts.

The accuracies and precisions obtained using the forearm mockup are consistent with those achieved in our previous work, although obtained with a larger number of magnets, viz. five instead of four [11]. The mean position accuracy $\bar{e_{m_{p}}}$ ($\bar{e_{ct_{p}}}$) demonstrated always lower than 0.22 (1.19) mm, while the mean precision $S_{m_{p}}$ ($S_{ct_{p}}$) proved below 120 (430) µm in all configurations, yielding to highly repeatable measurements (Figure 5b). The high linearity found between the real and computed displacement (Figure 5a) is important for direct control in a hand prosthesis, as it means that the system can preserve the relationship between muscle contraction and the relative movement in the prosthesis.

The higher sensibility exhibited by MM2, and for all configurations where this was used, is likely to be due to its distance to the sensor matrix. This effect was already reported in our previous works [11,18]. However, here we provide additional items to the discussion. First, the present result suggests that a wrong positioning of a magnet yields to inaccurate localizations for that magnet, regardless the number of magnets involved (Figure 5b). Second, while the wrongly positioned magnet is highly sensible to movements of the other magnets and to the variability associated to repeated measures, it is also true that its effect on the other magnets is negligible. Hence, in the case one magnet during the surgery was accidentally implanted too far from the AU, that magnet could be taken into account but simply ignored for control, without significant effects on the functioning of the remaining magnets.

The shorter computation times exhibited by the embedded platform are largely due to the code parallelization (using the DSP) and optimization (Figure 6). The absolute result—4 ms to localize the pose of five magnets—is rather promising in the perspective of the target application, where delays up to 100 ÷ 250 ms are considered acceptable [21,22]. Our result leaves an ample time margin that can be exploited to increase the number of implanted (and tracked) magnets, and thus to increase the DoFs controllable with the myokinetic approach. More specifically, if the goal is to control the five digits, including the abduction of the thumb, along with the pronation/supination and flexion/extension of the wrist, the localizer should be able to track up to 16 MMs. Theoretically, 32 three-axis sensors would be enough to retrieve their unknown position and orientation. In practice, to improve the accuracy of the LMA, more sensors, spatially distributed in proximity of the implanted MMs, would be needed. Although our results suggest the feasibility of such implementation from a computational viewpoint, timing issues deserve further analyses. Notably, an increase in the number of magnets and sensors implies not only a longer CT but also longer acquisition and data-transfer times (Figure 2). This to say that improving the performance of the system may require to modify its architecture.

In fact, the developed embedded system showed limitations under different aspects that curtail its use in an actual prosthesis. First, the sampling could not be accurately synchronized across sensors due to hardware limitations of the AU (and the architecture of the sensors). However, this represents a drawback concerning the consistency of the data and in turn the bandwidth of the localizer. Hence this study invites the development of an AU capable of simultaneous (triggered) sampling from a number of sensors. Second, the present implementation did not consider nor compensated for environmental factors, such as external magnetic fields or the effects of relative compression/depression movements between the socket (where the localizer is) and the stump tissues. These issues are known to degrade the performance of the localizer [11] and thus will be addressed in future studies. Third, the embedded system showed limited performance in terms of power efficiency. Overall it requires ~980 mW, which is a considerable amount of power for a (portable) prosthesis controller. Indeed, low power consumption is fundamental in a prosthesis, as larger batteries mean more weight the user has to carry, and less space available for the other components. Finally, it should be noted that while each component of the localizer represents a reasonable design choice, the architecture and the hardware of the embedded system could be optimized in order to take into account all the previous limitations.

With dedicated technical effort, a custom hardware (including both the sensing and computation apparatus) based on the CU (and, specifically, the CPU) used here could be developed and directly integrated into a prosthetic socket, making the system truly portable. This paves the way towards the development of a human-machine interface that allows simultaneous and proportional control of multiple movements of a hand prosthesis. Yet, potentially, this solution could be generalized to any type of upper or lower limb amputation, given that the muscles in the forearm represent the worst case due to their sizes and tightness.

## Figures and Tables

**Table 1:** Experimental conditions tested using the forearm mockup.

Configuration /No. of MMs	Mockup Muscles “Implanted”
1	Extensor carpi ulnaris
2	*Conf.* #1 + Extensor carpi radialis longus
3	*Conf.* #2 + Extensor carpi radialis brevis
4	*Conf.* #3 + Extensor digitorum communis
5	*Conf.* #4 + Extensor digiti minimi
